# Role of Decorin Core Protein in Collagen Organisation in Congenital Stromal Corneal Dystrophy (CSCD)

**DOI:** 10.1371/journal.pone.0147948

**Published:** 2016-02-01

**Authors:** Christina S. Kamma-Lorger, Christian Pinali, Juan Carlos Martínez, Jon Harris, Robert D. Young, Cecilie Bredrup, Eva Crosas, Marc Malfois, Eyvind Rødahl, Keith M. Meek, Carlo Knupp

**Affiliations:** 1 NCD-BL11, ALBA Synchrotron Light Source, Cerdanyola del Vallés, 08290, Barcelona, Spain; 2 Structural Biophysics Research Group, School of Optometry and Vision Sciences, Cardiff University, Cardiff, CF24 4HQ, United Kingdom; 3 Department of Ophthalmology, Haukeland University Hospital, 5021 Bergen, Norway; 4 Department of Clinical Medicine, University of Bergen, 5020 Bergen, Norway; University of Oklahoma Health Sciences Center, UNITED STATES

## Abstract

The role of Decorin in organising the extracellular matrix was examined in normal human corneas and in corneas from patients with Congenital Stromal Corneal Dystrophy (CSCD). In CSCD, corneal clouding occurs due to a truncating mutation (c.967delT) in the decorin (*DCN*) gene. Normal human Decorin protein and the truncated one were reconstructed *in silico* using homology modelling techniques to explore structural changes in the diseased protein. Corneal CSCD specimens were also examined using 3-D electron tomography and Small Angle X-ray diffraction (SAXS), to image the collagen-proteoglycan arrangement and to quantify fibrillar diameters, respectively. Homology modelling showed that truncated Decorin had a different spatial geometry to the normal one, with the truncation removing a major part of the site that interacts with collagen, compromising its ability to bind effectively. Electron tomography showed regions of abnormal stroma, where collagen fibrils came together to form thicker fibrillar structures, showing that Decorin plays a key role in the maintenance of the order in the normal corneal extracellular matrix. Average diameter of individual fibrils throughout the thickness of the cornea however remained normal.

## Introduction

The Extracellular Matrix (ECM) plays a key role in the maintenance and function of connective tissue and consists predominantly of collagen, proteoglycans, and other minor proteins [1]. Proteoglycans are macromolecules composed of a protein core and one or more carbohydrate glycosaminoglycan (GAG) side chains. GAGs are negatively charged, highly hydrophilic polysaccharides. The core protein of a proteoglycan can interact with collagen fibrils at specific sites [2,3]. These interactions are important in maintaining the overall structural organisation of the extracellular matrix. Therefore, any alterations in these molecules might perturb connective tissue stability.

Dermatan sulphate proteoglycans, of which the most common is Decorin, are small leucine rich proteins (SLRPs) with an “arc” shaped spatial conformation, which tend to form dimers within the tissue [4] and when forming crystals *in vitro* [5]. An interesting feature of SLRPs is that they contain an amphipathic consensus sequence, with leucine as the predominant hydrophobic residue placed in conserved positions [1]. This pattern was suggested to be involved in “protein-protein” or “protein-lipid” interactions [6]. The amino-acid sequence of these proteins is characterised by long arrays of leucine-rich repeat motifs of about 24 amino acids in length [4]. With respect to collagen/proteoglycan interactions several theories have been proposed to date, with the majority of them suggesting that interactions with collagen are established at the inner site of the “horseshoe” shaped molecule [7,8,9].

Bearing in mind that the concave surface is involved in a high-affinity dimer interaction, this theory has been debated [10,11] but no consensus was reached in view of the fact that there might be a dimer-to-monomer transition of proteoglycans in collagen/proteoglycan interactions [12]. This latter suggestion is further supported by the fact that Decorin is biologically active as a monomer in solution [13]. However, according to Scott et al., (2004) dermatan sulphate proteoglycans bind to collagen as a dimer and conservation analysis across class I SLRPs revealed a clustering of partially conserved residues on the sugar-free surface of LRRs IV-VI, a region that has been implicated in collagen binding. This, though, has yet to be confirmed.

Congenital stromal corneal dystrophy (CSCD) is associated with mutations in the Decorin gene (*DCN*). All reported mutations (c.967delT, c.962delA, c.947delG and c.941delC) are predicted to cause a 33 amino acid deletion in the C-terminus of the Decorin core protein [14, 15, 16, 17]. This condition is an autosomal dominant trait that is characterised by bilateral corneal opacities that are present shortly after birth [14]. Ultrastructural evaluation of affected corneas using electron microscopy revealed areas of seemingly normal corneal stroma interrupted by areas in which the regular arrangement of collagen fibrils was disrupted. Regions of amorphous substance containing abnormal filaments were intermittently seen at the interface between the ordered and disorder areas in the stroma [14, 16, 18, 19, 20]. The truncated Decorin is found to aggregate *in vivo* and it is found to accumulate in these interlamellar areas that are characteristic of CSCD [21]. In the present study we wish to ascertain whether the truncating mutation has affected Decorin’s ability to form stable dimers or to establish the appropriate interaction with collagen fibrils.

In this study, we use electron microscopy 3-D tomography and Small Angle X-ray diffraction (SAXS) to examine the extracellular matrix in CSCD. We also carry out homology modelling to obtain a 3-D model structure of the core protein of Decorin and we investigate how this is affected by the c.967delT CSCD mutation.

## Materials and Methods

### Homology modelling

The homology modelling protocol was described by Dalton and Jackson (2007) [22]. In brief, a BLAST search was performed in EXPASY to find proteins with the highest sequence similarity to the human Decorin protein. Bovine Decorin was indicated as the highest match with a similarity in the two sequences of 82% [5]. ClustalW was applied for structural similarities between the two proteins. Secondary structure prediction was performed using the Expasy application.

Modweb application in Modeller [23], was used to construct the initial model. Energy minimisation was performed initially in Chem 3D ultra 12.0 [24]. After this the structure was transferred to a LINUX workstation where a Molecular Dynamics simulation was performed using Gromacs software [25]. Hydrogen molecules were added to the molecule to neutralise the charges and the protein was placed in a box containing water molecules where a steepest descent energy minimisation algorithm was applied for 200ps at 300K in 2ps steps. Subsequently, a Low-memory Broyden-Fletcher-Goldfarb-Shanno quasi-Newtonian minimiser (L-BFGS) was applied till 0.15 kcal/mol/Å was reached, in order to correct the Newtonian trajectories.

It has to be noted that the constructed model included residues 51–355 of the unprocessed Decorin gene product (corresponding to residues 22–325 of the mature protein) as the crystal structure of bovine Decorin that was used as a template was missing the same parts due to lack of interpretable electron density caused by conformational disorder [5]. The rest of the template candidates that were provided by the BLAST search were also missing these parts of the protein.

### Tissue samples and preparation

All human tissue was obtained with patient written consent according to the tenets of the Declaration of Helsinki and after approval of the local ethical regulations (i.e. Regional Committee for Medical and Research Ethics, Western Norway IRB #00001872). Two CSCD corneal samples were obtained after corneal transplantation was performed in patients with CSCD carrying the c.967delT mutation and processed for electron microscopy. One cornea was plunge frozen and stored in liquid nitrogen until it was used for electron microscopy. The other one was transported in culture medium containing 1% Dextran and was used for SAXS experiments. Two normal human corneas were obtained from the Bristol Eye Bank (UK) and the Northwest Lions Eye Bank (USA), after donor consent and ethical approval. One of the normal corneal samples was treated with Chondroitinase ABC enzyme as previously described [26] and the other one served as control. The CSCD sample was thawed and all tissue samples were dissected into smaller pieces that were then fixed overnight in 2.5% glutaraldehyde in 25 mM sodium acetate buffer, pH 5.7, containing 0.1 M magnesium chloride and 0.05% cuprolinic blue at conditions of ‘critical electrolyte concentration’ appropriate to demonstrate all proteoglycans. The samples were then briefly washed in buffer followed by 15 min incubations in aqueous 0.5% sodium tungstate and subsequently in 50% ethanolic 0.5% sodium tungstate. Specimens were then dehydrated through a series of increasing ethanol concentrations and finally they were embedded in Araldite resin and polymerized at 60°C for 48 hr. Ultrathin sections (~100nm thick) were stained with 1% phosphotungstic acid and saturated uranyl acetate solutions, and examined using a JEOL 1010 (EM208; Philips, Eindhoven, The Netherlands) transmission electron microscope at 80kV.

### EM tomography

Electron-tomography data were taken in a tilt series ranging between -60° and +60° in 1° increments for longitudinal view and 2° increments for transverse ones at 20k magnification, using a JEOL 1010 (JEOL U.K. Ltd, Welwyn Garden City, UK) transmission electron microscope at 80kV. The images were aligned using the IMOD software package [27] in Linux Ubuntu environment and using individual gold fiducial markers as a reference for the alignment. Finally, segmentation and 3-D reconstruction was performed using ImageJ [28] and EM3D [29] programs. In addition, the axial periodicity of aggregated protein bodies in the corneal stroma was measured using the “straighten” plug-in and profile plotting function in Image J.

### Small Angle X-ray diffraction (SAXS)

SAXS data was collected in NCD beamline, ALBA synchrotron light source, Barcelona, Spain. Part of a CSCD affected cornea that measured about 1/5 of the original removed tissue, was donated after corneal transplantation. The sample was transferred at 4°C and stored in culture medium that contained Dextran to prevent swelling. During X-ray data collection the sample was wrapped in plastic membrane and was placed between Mylar windows to prevent dehydration. Measurements were taken stepwise in a 2D matrix fashion covering the whole area of the tissue. X-ray data points were collected every 250 microns in horizontal and 500 microns in vertical directions. The X-ray beam energy was at 12.4 keV and the sample to detector distance was 6.25 meters. Data analysis that revealed averaged fibrillar diameters throughout the thickness of the tissue at each measured point was performed as previously described [30,31].

## Results

### Homology molecular modelling

**Fig 1:**
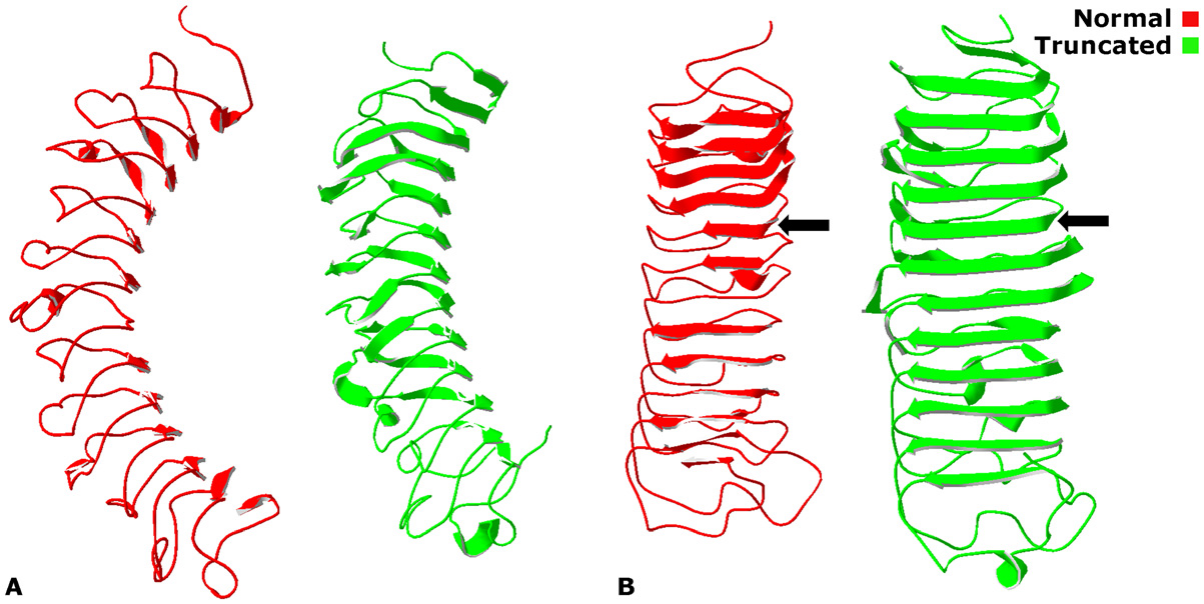
Homology 3-D models for normal and truncated Decorin. In the c.967delT decorin model serine was replaced with leucine at position 323 and this caused the mutant protein to be shorter than the normal one, as it stops at position 327. The truncated protein is less curved (A) with wider β-sheets in the inner cavity (B) than the normal one. β-sheets in normal and truncated protein are indicated with black arrows.

The molecular modelling experiments of the c.967delT mutant have shown that truncated Decorin appears to be less curved (Fig 1A) with wider β-sheets in the inner cavity (Fig 1B) than normal Decorin, and it seems that the protein is trying to space out its residues to compensate for the shorter primary structure. As the mutation causes a truncation in the C-terminus [14] of the protein, the sole GAG binding side in the N-terminus [5] is still intact.

**Fig 2:**
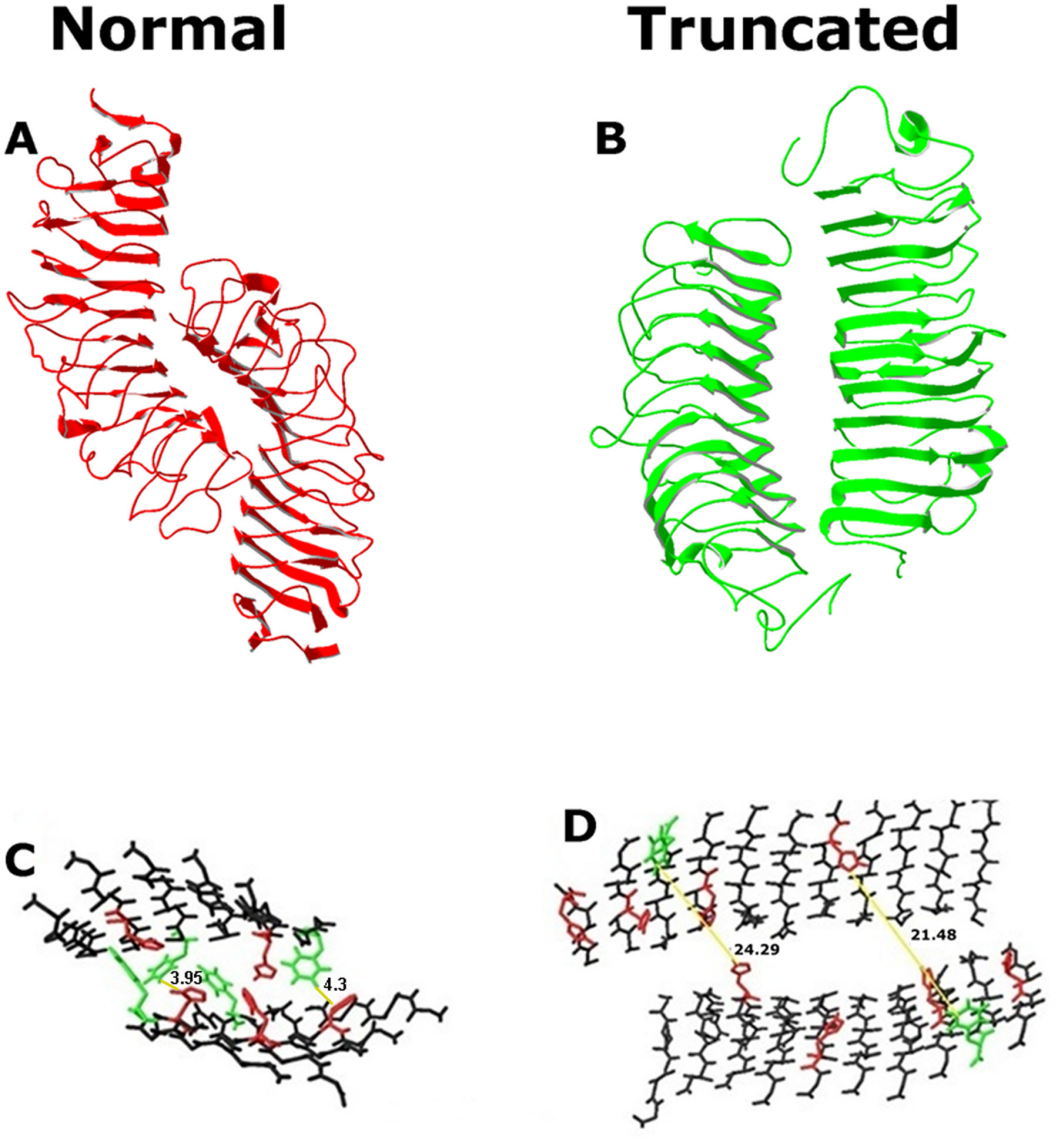
Comparison of the dimer concave interface in normal and truncated Decorin. The mutation not only affected the structure of Decorin, but it also changed its ability to form stable dimers able to interact with collagen. The geometry and the spatial arrangement of the mutant are different from the normal one (A and B). The concave surface in both the normal and mutant Decorin form the dimer interface. In normal Decorin hydrophobic interactions are shown to be established predominantly between the aromatic rings of phenylalanine (green) and histidine (red) (C) [5]. In the truncated model, the concave surface is much less curved, the dimer interface consists of more β-sheets than the normal, and the two proteins are further apart (D).

The truncating mutation in Decorin seems to affect the spatial arrangement of the protein (Fig 2A and 2B). The dimerisation occurs between the concave parts of the protein [5], where hydrophobic bonding between Phenylalanine (Phe-green colour in Fig 2) and Histidine (His-red colour in Fig 2) stabilises the dimer formation in normal Decorin (Fig 2C). In the truncated protein this bond is disrupted (Fig 2D) and this increases the distance between the two proteins making the available Phe residues >20Å apart from the nearest His residues in the opposite monomer. This probably affects the establishment of the expected hydrophobic interaction as the distance of this specific bond is known to be at around 4Å [32,33]. Hence, the stability of the truncated Decorin dimer may depend on very weak hydrophobic interactions or other type of bonding. It is also possible that dimers in CSCD mutant Decorin are formed only transiently and do not play a defined structural role.

### Electron 3-D Tomography and SAXS

**Fig 3:**
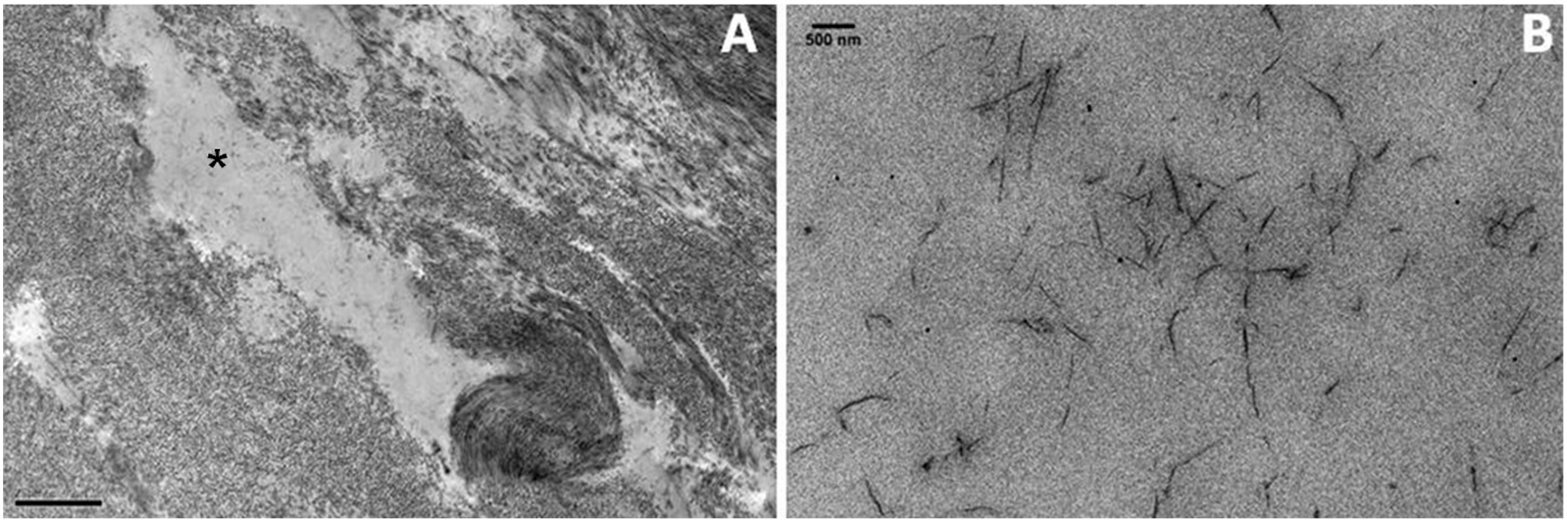
Appearance of stromal “spaces” in the C967delT mutant cornea. Collagen in the corneal stroma was often disorganised with the formation of “spaces” (asterisk in A). These regions contained oversized proteins that are believed to be aggregated proteoglycans that fail to interact with collagen (B).

Electron microscopy of CSCD corneas has shown both apparently normal and disorganised regions in the corneal stroma, as the mutation is a heterozygous one and hence affects half the Decorin protein content [14, 18]. Areas of disorganised collagen in the corneal stroma in the human c.967delT CSCD sample were examined (Fig 3A). In stromal “spaces” that were formed between the lamellae (asterisk in F 3A) there were amorphous filaments (Fig 3B).

**Fig 4:**
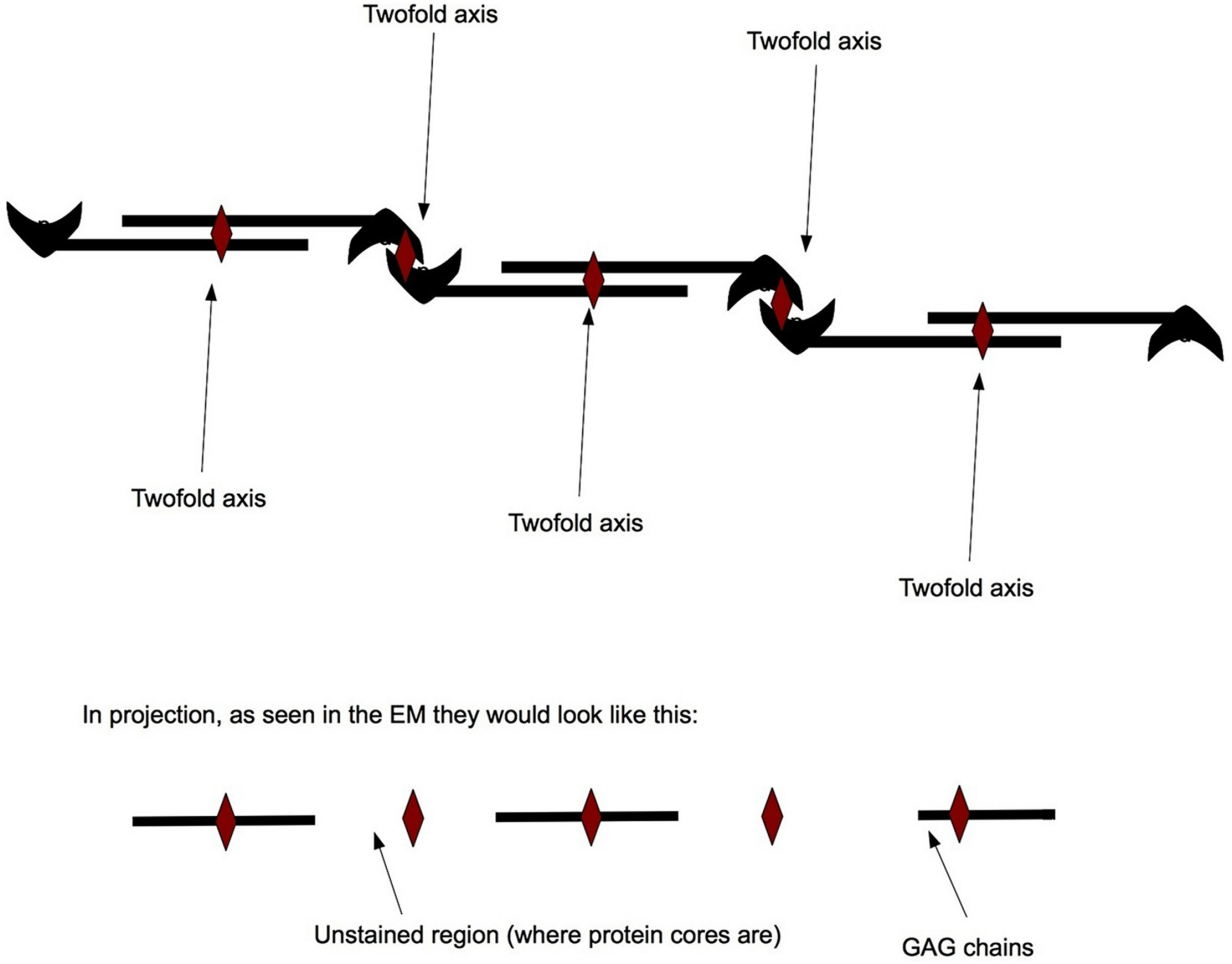
Theoretical representation explaining protein aggregation in stromal “spaces”. The mutation in Decorin in the case of CSCD did not affect the protein’s ability to form dimers, but prevented it from interacting with collagen and caused it to subsequently aggregate in lamellar gaps in the diseased stroma. The aggregated molecules formed anti-parallel associations. The regions with the protein cores are unstained and invisible, whereas the two fold axes are where the red diamonds are.

We postulate them to be large proteoglycan bodies which have aggregated as a consequence of the structural change of Decorin caused by its truncating mutation. In many cases these aggregates seemed to have a regular arrangement with varying axial periodicity. Electron dense regions were separated by unstained parts measuring 8.45 nm (±1.2 nm) in length. The theoretical model describing the truncated Decorin measured ~8.36 nm as a dimer and ~6.18 nm as a monomer. Therefore, it is likely that Decorin still forms dimers in CSCD, but these dimers might be unable to interact effectively with collagen. However, anti-parallel aggregates are still able to form and are those seen in the “spaces” with the electron dense parts being the GAG chains and the unstained parts being the Decorin dimers (Fig 4).

**Fig 5:**
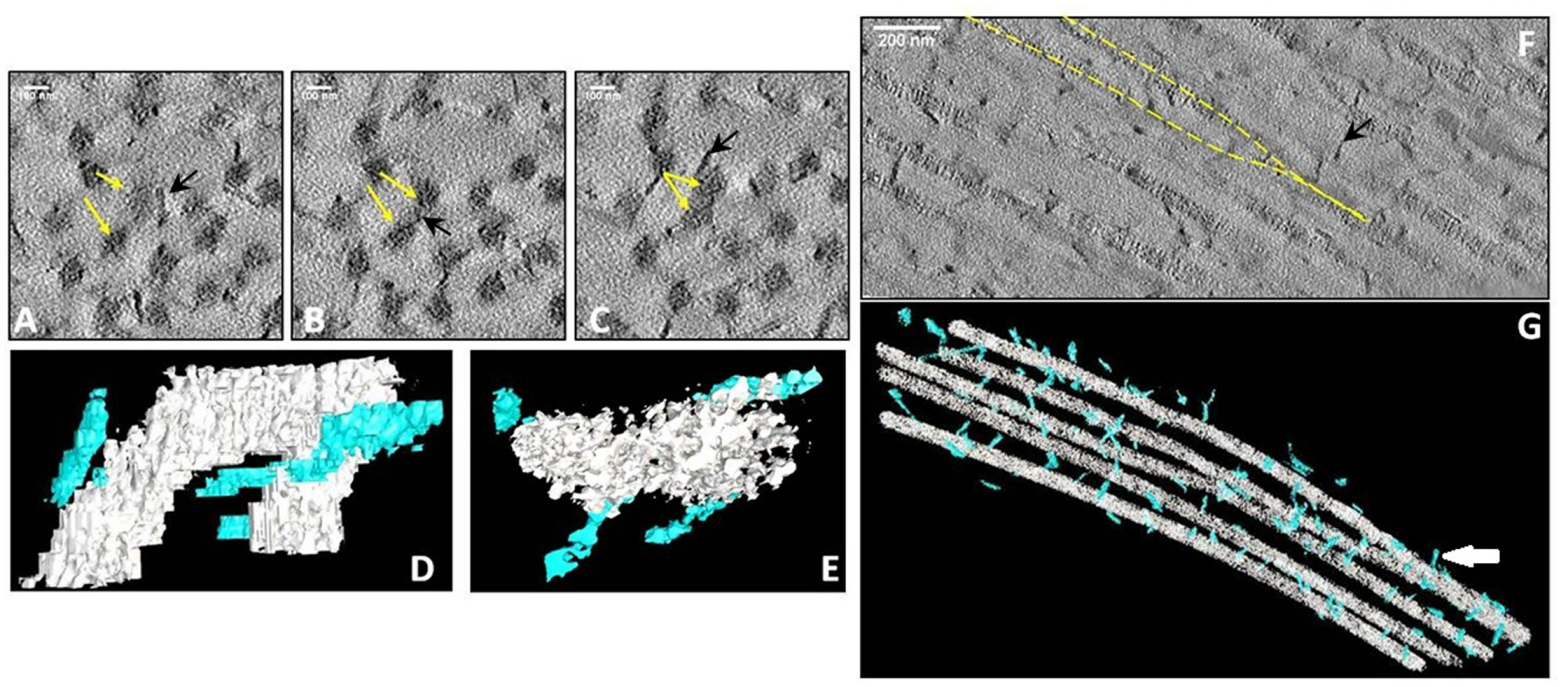
3-D reconstruction showing collagen fusing in areas of abnormal lamellae in CSCD. Sections through 3-D reconstruction showing two collagen fibrils (yellow arrows) coming together as we move through the depth of the CSCD sample (A-C). 3-D segmentation of these two fibrils in longitudinal (D) and transverse (E) view. Longitudinal section through a 3-D reconstruction showing the association of separate collagen fibrils (yellow dashed line) in the corneal stroma (F). 3-D segmentation of a wider part of the same stromal area showing the association of fibrils (white arrow in G). In electron micrographs yellow arrows indicate collagen fibrils and black arrows proteoglycans (A, B, C, F). In 3-D electron tomography collagen is depicted in white and proteoglycan chains in blue-green (D, E, G).

Due to the presence of dysfunctional Decorin, there were some regions in the stroma where collagen was disorganised and fibrils were coming together to form thicker bundles (S1 Movie). Fig 5A–5C shows successive sections through a 3-dimensional reconstruction showing two collagen fibrils coming together (yellow arrows). These fibrils were segmented and are shown in white in Fig 5D and 5E along two different view-points (S2 and S3 Movies). Proteoglycans are shown in blue-green. Fig 5F shows a section through a 3-D reconstruction of collagen fibrils in longitudinal view. Two fibrils joining together are highlighted in yellow. These can clearly be seen in the segmentation in Fig 5G (white arrow). However, SAXS experiments demonstrate that average individual fibril diameter remains normal 35.88 ±0.4 nm (standard deviation of mean).

**Fig 6:**
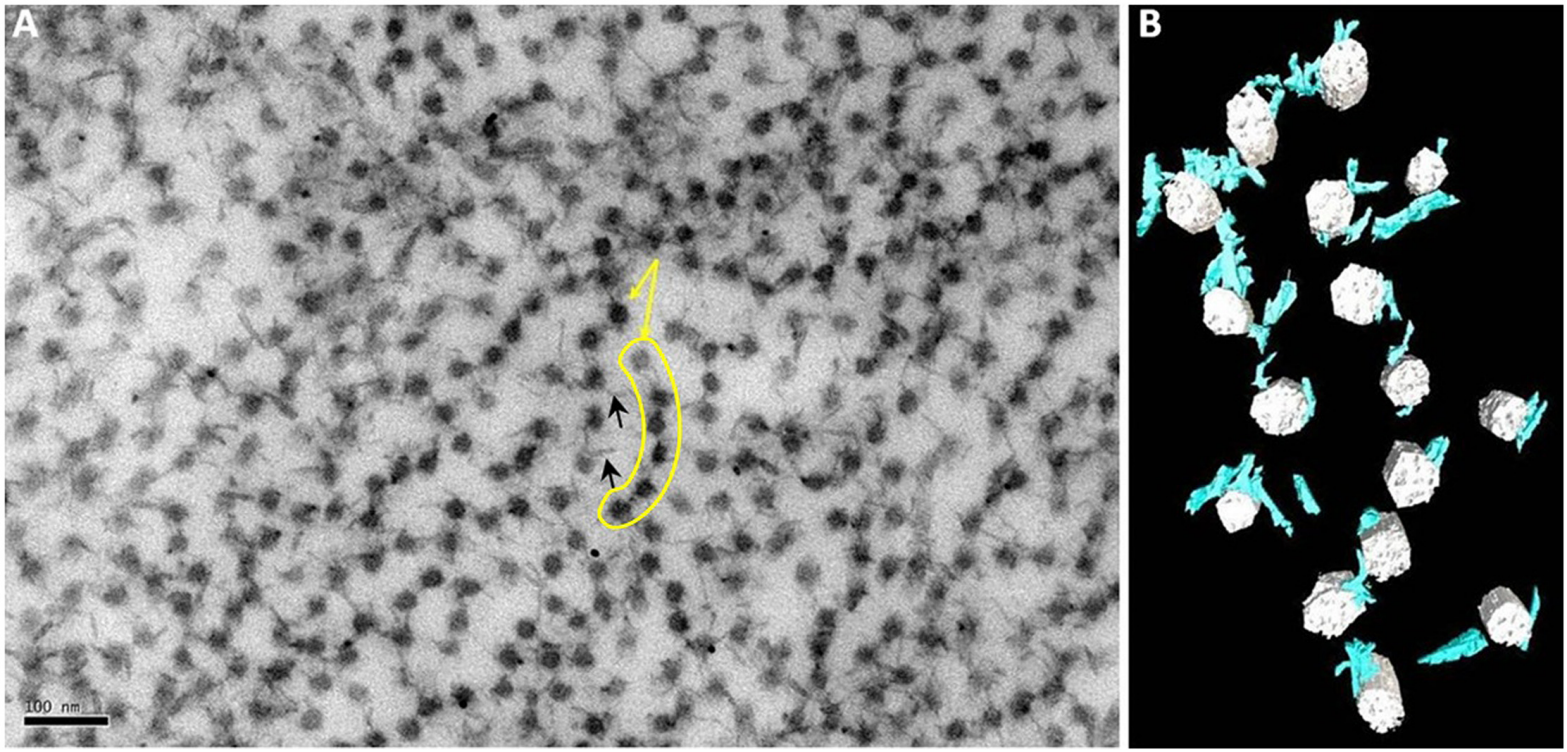
3-D reconstruction of collagen fibrils that lost the regular arrangement observed in the normal cornea forming necklace-like arrangements. In the disorganised matrix that was observed in CSCD areas where collagen fibrils formed necklace-like arrays were present (yellow arrows and highlighted area in A). Proteoglycan chains linked the fibrils together (B). In the electron micrograph, yellow arrows indicate collagen fibrils whereas black ones indicate proteoglycans in the corneal stroma.

In areas where there were no large stromal “spaces”, collagen fibrils had often lost the regular arrangement that is observed in the normal cornea. Instead, shorter proteoglycans joined arrays of fibrils together forming “necklace-like” structures (Fig 6). One of these structures is highlighted in the electron micrograph in Fig 6A and with a segmentation shown in Fig 6B and in S4 Movie.

### Chondroitinase digestion

**Fig 7:**
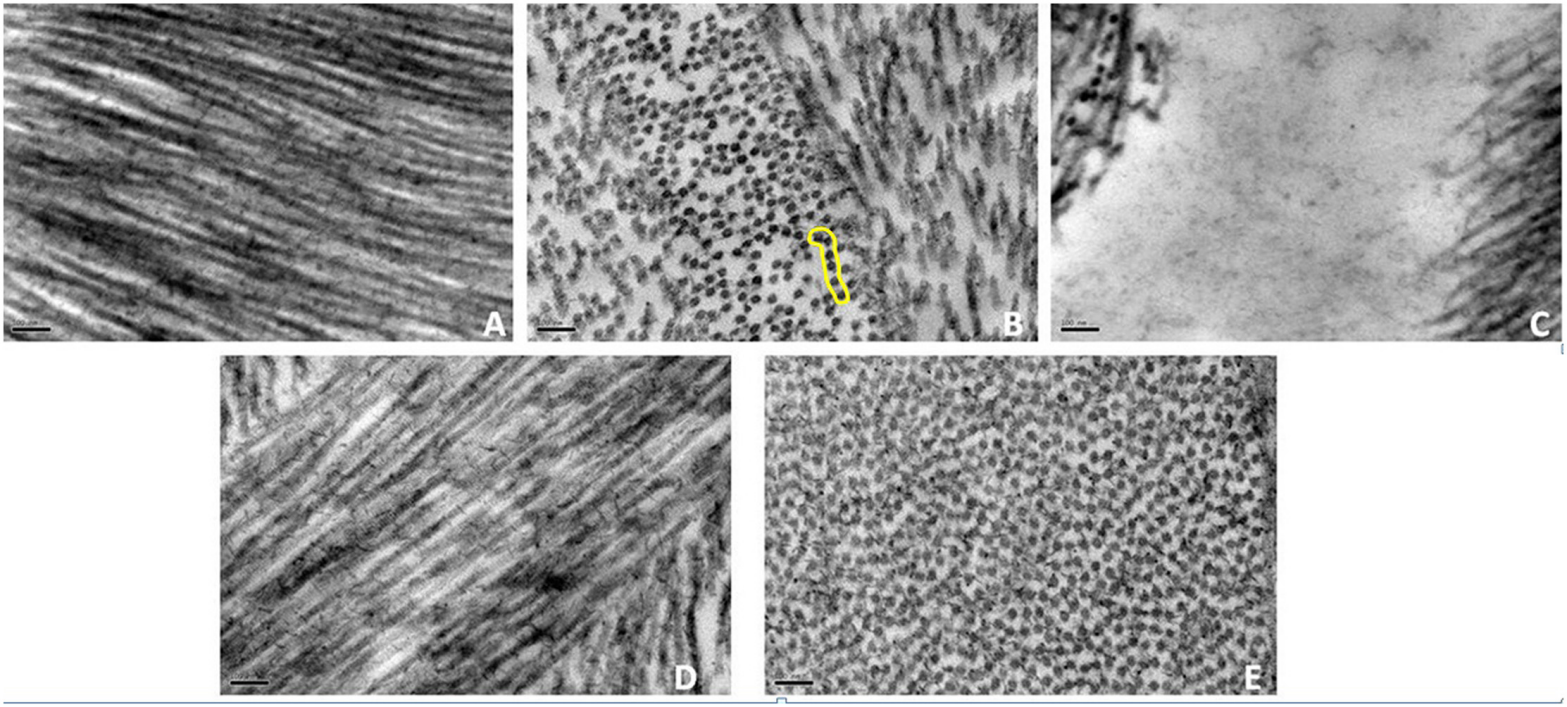
Chondroitinase enzyme digestion removed completely the CS/DS content of the tissue and cause similar effects to CSCD in the cornea. A normal human cornea treated with Chondroitinase enzyme presented similar characteristics as the sample with CSCD. Fibrils were disorganised and associating (A), forming necklace-like structures (highlighted by a yellow line in B) and collagen lamellae separated to give rise to “spaces” (C). Normal human cornea without any enzyme treatment (D and E).

A normal human cornea that was treated with Chondroitinase ABC enzyme appeared to have similar collagen organisation as the one observed in the disorganised regions of the CSCD sample. The complete removal of Chondroitin/Dermatan Sulphate (CS/DS) proteoglycans caused a structural breakdown within the tissue, which then appeared very different from the normal untreated sample (Fig 7). Fig 7A–7C show Chondroitinase ABC enzyme treated corneas from which the CS/DS proteoglycans had been removed in longitudinal (Fig 7A) and transverse section (Fig 7B). The stroma was disorganised and areas (i.e. “spaces”) that contained aggregated proteins were seen (Fig 7C). Fibrils in this cornea were seen to come together to form thicker bundles. In many places necklace-like structures were formed (highlighted in Fig 7C). Fig 7D and 7E shows normal untreated human cornea for visual comparison.

## Discussion

The aim of this study was to examine the role of Decorin in the structural disruption of the tissue in CSCD to improve our understanding of the role of Decorin in collagen arrangement and organisation of the extracellular matrix. Previous genetic analyses in affected individuals have shown that the disorder is associated with truncating mutations in the Decorin gene [14, 15, 16, 17, 18]. All these mutations are predicted to activate the same stop codon, causing the protein to be truncated with 33 amino acids missing compared to the wild type.

Previous transmission electron microscopic examination of CSCD corneal samples has shown regions of normal stroma disrupted by amorphous areas “spaces” with small proteoglycan filaments that disturb the stromal architecture [14, 16, 18, 19, 20]. In addition, the “spaces” were found to contain scattered proteoglycan molecules that showed intense labelling for proteoglycan chains. The truncated Decorin was found to aggregate [21]. A closer examination in the current study using 3-D electron tomography showed an axial association of proteoglycans leading to the formation of abnormal structures in these regions, suggesting that there is an aggregation of dermatan sulphate PGs in the dystrophic corneal stroma (Figs 3B and 4). This was supported by our molecular modelling studies that indicated that in CSCD, the structure of Decorin core protein is compromised.

**Fig 8:**
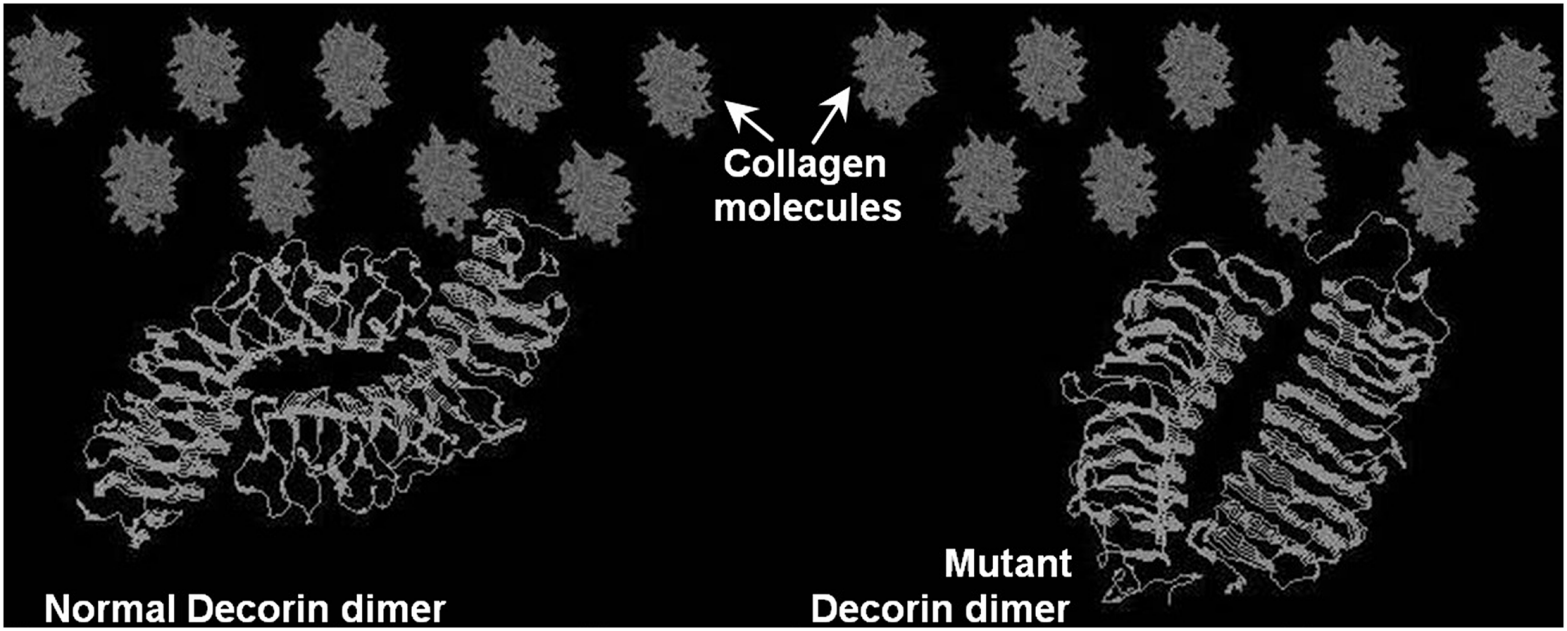
Observation of the mutant dimer’s ability to interact with collagen. Manual docking representation for truncated and normal Decorin dimer. The collagen molecules represent part of a fibril. Reduced size of the mutant protein limits its association with collagen.

The structure of CSCD decorin core protein that was obtained by applying homology modelling techniques, appears much smaller than normal, and this potentially can impair the protein’s ability to interact and bind effectively to collagen. Descent from a common ancestor, i.e. homology, can be hypothesised when similar properties are detected in biological structures [34]. Comparative homology-based modelling used in this study is based on the observation that sequence correlation above a certain proportion implies structural similarity. Thus, a protein of a known structure and sequence can be used as a template for the construction of a 3-D model of another protein with a sequence similar to the template [35]. Additionally, it should be noted that structure is conserved to a much greater extent than sequence, and that there is only a limited number of possible backbone motifs [36,37]. In order to ensure the construction of a reliable model, the minimum percentage of homology between two protein sequences should be ~40% [38]. Template and model homology sequence identity greater than 50% normally have 90% or more of the individual protein structures in common, and a Root Mean Square Deviation (RMSD) value smaller than 1Å, indicating the reliability of the proposed model [39]. Once both normal and truncated homology models have been obtained, they were manually docked to collagen. It was evident that the truncated dimer could not reach the collagen molecules appropriately because of its limited size and the proteins did not seem to be able to establish sufficient interactions (Fig 8).

Selective digestion of normal human corneal stroma with Chondroitinase ABC, removes chondroitin sulphate side chains and hence compromises the formation of proteoglycan networks and binding to collagen within the tissue. This induced similar effects to those seen in the CSCD cornea supporting the view that the truncating mutation in Decorin that prevents it to binding to collagen is likely to be the major reason for the structural disorganisation of the tissue.

It has been suggested that Decorin interacts with collagen as a dimer [5, 40]. An axial one-dimensional analysis of the aggregated structures in the “spaces” has shown that the unstained regions between the GAG chains in electron micrographs appear to be of similar size to the truncated dimer model that we obtained in our homology modelling experiment. This supports the idea that Decorin is a major component of these filaments and that it is still able to assemble as dimers, but these dimers are not able to form stable interactions with collagen fibrils. The long filamentous aggregates seen in the “spaces” in the CSCD cornea were consistent with a structure comprising anti-parallel associations of proteoglycans with Decorin protein cores. The GAG chains, being highly charged, would be stained and show prominently in the electron micrographs. The Decorin protein cores, being less charged, would be poorly stained and appear as gaps. Both protein cores and GAG chains would interact in an anti-parallel manner (protein cores with protein cores and GAG chains with GAG chains-Fig 4) leading to the formation of long filamentous structures. The amount by which the GAG chains overlap could vary depending on the sulphation of the chains (for example, if their association was mediated by positive free ions forming salt bridges with both chains) therefore keeping the chains together.

According to a peptide segment analysis for human Decorin there are two main domains that interact with collagen type I [41]. Amongst those, the one between residues 185–329 has the highest affinity for collagen type I [42]. The point mutation in this particular study stops the amino acid sequence at residue 319 reducing the size of the protein’s binding site. This also impairs the normal geometry of the protein making it much shorter. The truncating mutation does not affect the GAG binding site in the N-terminus, it only affects the end part of the protein (C-terminus), where the protein is believed to have a high-affinity collagen binding area in bands d and e1 [42]. Additionally, comparing Decorin proteins with point mutations that do not affect the overall size of the protein with truncated ones showed that the smaller sized Decorin truncated close to the C-terminus has the least affinity to form interactions with collagen type I [43]. Overall, our findings suggest that the c967delT mutation does not disrupt the entire function of the protein since it is still able to interact with other GAGs, but it has compromised Decorin’s ability to establish fibrillar interactions because of the protein’s partial cleavage of its binding site as well as its altered 3-D geometry. The truncated form of Decorin cannot interact with collagen in CSCD, but it would still be able to form aggregates between the fibrils or in the stromal “spaces”, possibly leading to the appearance seen in the electron microscope.

Another observation in this study was the fact that collagen fibrils, in some stromal regions, had lost the regular arrangement of the normal human cornea [44] and formed arrays that resembled a “necklace-like” structure when viewed in cross-section. The same effect was observed when bovine corneal samples were treated with enzymes removing CS/DS GAG chains [26] and in the enzyme-treated human cornea in this study (Fig 7). In CSCD-affected cornea the effect is probably due to a structural compromise of Decorin that leads to a very limited capability of this protein to associate with collagen, leaving only the KS proteoglycans to form connections between collagen fibrils as seen in the enzyme treated corneas. This also supports the suggestion on the roles of various proteoglycan types in the arrangement of collagen in the extracellular matrix [26, 45]. In particular, it was proposed that CS/DS proteoglycans, such as Decorin in our study, form longer chains that extend among several collagen fibrils and hence help to organise collagen in the extracellular matrix in a pseudo hexagonal arrangement, which is an essential property for maintaining transparency in the cornea [26]. On the other hand, KS form shorter chains, which seem to join adjacent collagen fibrils. In the case of CSCD affected cornea, the serious compromise in the function of Decorin has caused the corneal stroma in places to lose its regular collagen arrangement, whereas fibrils were still connected to each other by the KS proteoglycans forming “trains” or “necklace-like” structures. Sometimes collagen fibrils were seen to come together creating thicker, irregularly shaped fibrils. This is probably because Decorin’s protein cores are not binding to the collagen fibrils and hence they do not prevent adjacent collagen fibrils from coming into contact.

Average fibrillar diameter throughout the CSCD affected tissue remains within the normal range [46]. This is not in accordance with recent microscopy studies that found abnormally thinner collagen fibrils in disorganised stroma in CSCD corneas with a different truncating mutation [20]. However, microscopy techniques often sample just a small part of the tissue and hence, where certain features might be localised, observations don’t always represent the situation within the overall tissue. On the other hand, X-ray diffraction techniques require no previous sample preparation and hence internal structures are not affected by processing methods and in addition they allow us to examine a bigger sampling area within the tissue and thus have a wider idea of overall properties. This method provides us with an average of fibrillar diameter throughout the thickness of the cornea in each sampling point. Therefore, with the current findings we can assume that a major proportion of collagen fibrils appear to have normal diameters in the examined CSCD corneal sample due to averaged sampling of intermittent normal and abnormal lamellae throughout the thickness of the tissue, without excluding the existence of thinner fibrils.

Finally, all these findings on the failure of the truncated Decorin to organise the matrix in the CSCD cornea were cross validated and compared with enzyme digestion that was performed in a normal human cornea, using Chondroitinase ABC. It has to be noted that the enzyme digestion has removed entirely the CS/DS glycosaminoglycan content from the corneal stroma, whereas the truncation affects only 50% of the Decorin core protein content in the CSCD cornea as it is a heterozygous mutation [14]. However, areas of abnormal lamellae that were examined closely in this study appeared to have very similar characteristics to the ones observed in the enzyme treated cornea and this suggests that the structural compromises that were noted previously in CSCD are likely to be due to the malfunction of Decorin. In addition, in both the enzyme treated sample and the areas of abnormal lamellae that were examined in the CSCD cornea, all big proteoglycans had disappeared confirming that they are dermatan sulphate, as was previously proposed by Lewis et al., (2010).

In conclusion, truncated Decorin seems to form anti-parallel dimeric associations in CSCD affected cornea, but the dimers are not able to bind to collagen effectively. This causes disruptions of the normal corneal structure. In addition, abnormal filaments, presumably made of proteoglycans with affected Decorin protein cores, are seen to gather in stromal “spaces”. Our evidence suggests that this is aggregated Decorin. Adjacent collagen fibrils are seen to come together probably because of the absence of Decorin on their surface acting as a steric hindrance. Structural alterations of the cornea, the presence of “spaces” and the disordered arrangement of collagen fibrils, prevent effective transmission of the light travelling through the cornea causing corneal cloudiness.

## Supporting Information

S1 Movie Electron micrograph series throughout the thickness of the tissue showing fussion of fibrils. (AVI)

S2 Movie 3-D reconstruction showing fusion of collagen fibrils in transversal view. (AVI)

S3 Movie 3-D reconstruction showing fusion of collagen fibrils in longitudinal view. (AVI)

S4 Movie 3-D reconstruction showing “necklace-like” arrangement of collagen fibrils in CSCD. (AVI)
